# Testosterone Deficiency Is a Risk Factor for Severe COVID-19

**DOI:** 10.3389/fendo.2021.694083

**Published:** 2021-06-18

**Authors:** Lukas Lanser, Francesco Robert Burkert, Lis Thommes, Alexander Egger, Gregor Hoermann, Susanne Kaser, Germar Michael Pinggera, Markus Anliker, Andrea Griesmacher, Günter Weiss, Rosa Bellmann-Weiler

**Affiliations:** ^1^ Department of Internal Medicine II, Innsbruck Medical University, Innsbruck, Austria; ^2^ Central Institute for Medical and Chemical Laboratory Diagnosis, Innsbruck University Hospital, Innsbruck, Austria; ^3^ MLL Munich Leukemia Laboratory, Munich, Germany; ^4^ Department of Internal Medicine I, Innsbruck Medical University, Innsbruck, Austria; ^5^ Department of Urology, Innsbruck Medical University, Innsbruck, Austria

**Keywords:** testosterone, estradiol, inflammation, COVID-19, SARS-CoV-2, disease severity, outcome

## Abstract

**Background:**

Male sex is related to increased COVID-19 severity and fatality although confirmed infections are similarly distributed between men and women. The aim of this retrospective analysis was to investigate the impact of sex hormones on disease progression and immune activation in men with COVID-19.

**Patients and Methods:**

We studied for effects of sex hormones on disease severity and immune activation in 377 patients (230 men, 147 women) with PCR-confirmed SARS-CoV-2 infections hospitalized at the Innsbruck University Hospital between February and December 2020.

**Results:**

Men had more severe COVID-19 with concomitant higher immune system activation upon hospital admission when compared to women. Men with a severe course of infection had lower serum total testosterone (tT) levels whereas luteinizing hormone (LH) and estradiol (E_2_) levels were within the normal range. tT deficiency was associated with elevated CRP (rs = - 0.567, p < 0.001), IL-6 levels (rs = - 0.563, p < 0.001), lower cholesterol levels (rs = 0.407, p < 0.001) and an increased morbidity and mortality. Men with tT levels < 100 ng/dL had a more than eighteen-fold higher in-hospital mortality risk (OR 18.243 [95%CI 2.301 – 144.639], p = 0.006) compared to men with tT levels > 230 ng/dL. Moreover, while morbidity and mortality showed a positive correlation with E_2_ levels at admission, we detected a negative correlation with the tT/E_2_ ratio upon hospital admission.

**Conclusion:**

Hospitalized men with COVID-19 present with rather low testosterone levels linked to more advanced immune activation, severe clinical manifestations translating into an increased risk for ICU admission or death. The underlying mechanisms remain elusive but may include infection driven hypogonadism as well as inflammation mediated cholesterol reduction causing gonadotropin suppression and impaired androgen formation. Finally, in elderly late onset hypogonadism might also contribute to lower testosterone levels.

## Introduction

Coronavirus disease 2019 (COVID-19) is still influencing the daily life of people all over the world. Most patients infected with the severe acute respiratory syndrome coronavirus 2 (SARS-CoV-2) have developed mild symptoms, while up to 20% need hospitalization due to severe disease course characterized by shortness of breath and hypoxia (1). Several comorbidities including hypertension, obesity, diabetes, cardiovascular disease, and chronic pulmonary disease as well as age were shown to be related to more severe COVID-19 courses (2–5). Additionally, male sex is related to more severe COVID-19 manifestation, even though there are no sex differences in the absolute number of confirmed COVID-19 cases (6). Currently, up to 60% of hospitalized patients and even up to 82% of patients treated in the intensive care unit (ICU) are men (3, 4, 6). Accordingly, the case fatality rate (CFR) is 1.7 times higher in men compared to women (6–8). Inflammatory biomarkers were shown to be associated with COVID-19 morbidity and mortality in men and women: interleukin 6 (IL-6) as main inducer of C-reactive protein (CRP) in the liver (9), neopterin reflecting macrophage activation and thus T-helper cell type I (Th1) immune response (10) as well as other acute phase proteins including procalcitonin (PCT) and ferritin (11).

Several reviews have elucidated potential mechanisms underlying these sex differences in COVID-19 patients and will be only shortly summarized hereafter (6, 8, 12). Biological sex affects immune responses to invading pathogens through hormonal regulation, gene expression and environmental factors (8). Generally, women have a stronger innate and adaptive immune response than men resulting in faster pathogen clearance (13). Many genes located on the sex chromosomes regulate innate immune function by encoding for pattern recognition receptors, cytokine receptors or transcriptional factors (14). On the other hand, androgen response elements and estrogen (E_2_) response elements are found in promoters of several innate immunity genes thereby affecting their expression (15, 16). These mechanisms are suggested to contribute to faster containment and clearance of SARS-CoV-2 in female patients (8). Moreover, sex-associated differences in expression and activity of the virus entry receptor angiotensin-converting 2 (ACE2) and its co-receptor transmembrane protease serine subtype 2 (TMPRSS2) are suggested to contribute to a higher mortality in men (17, 18). The ACE2 gene is located on the X chromosome and estrogens were shown to upregulate its expression (19), while TMPRSS2 was shown to be regulated by androgen receptor signalling (20–22). Preclinical studies of ACE2 tissue expression have shown different results depending on the tissue type (23): ACE2 expression seems to be higher in the lungs, heart or kidney of male (24–26), while pancreatic ACE2 expression seems to be higher in female (27). However, the relationship of tissue ACE2 expression and circulating ACE2 activity is still not well understood and data from the literature is partial contradictory (23): in human, the ACE2 activity was shown to be higher in healthy men and men with heart failure compared to matched women (28, 29) while other studies showed no sex-related differences in serum ACE2 activity (30, 31). Interestingly, ACE2 activity does not differ between young and old men but is significantly higher in older compared to younger women (31).

However, whether these mechanisms actually affect disease severity and clinical outcome of COVID-19 patients or explain the sex differences remains unknown. Therefore, we analyzed different sex hormones and their interactions with inflammatory markers to assess the impact on disease severity and outcome in men: the androgen testosterone is the primary male sex hormone playing a key role in the male reproductivity and produced by testicular Leydig cells upon stimulation by luteinizing hormone (LH) released by the pituitary gland and regulated by the gonadotropin-releasing hormone (GnRH) released by the hypothalamus (32). Estradiol (E_2_) is the primary female sex hormone but also involved in male reproduction and synthesized from androgens by aromatase (33). Sex hormones are transported in the human body unbound in the serum or bound to the sex hormone binding protein (SHBG) (32).

## Material and Methods

### Study Population

We analyzed 377 patients with polymerase chain reaction (PCR)-proven COVID-19 hospitalized at our department at the Medical University of Innsbruck, Austria, between February and December 2020. Information on past medical history, concomitant medication, clinical characteristics and laboratory parameters were obtained from the local clinical electronic data management. The severity of the disease was categorized according to the score by the WHO Working Group on the Clinical Characterization and Management of COVID-19 infection (34). Events during hospital stay including death, ICU admission and need for mechanical ventilation were recorded for outcome analysis.

The study was conformed to the principles outlined in the Declaration of Helsinki and was approved by the ethics committee of the Innsbruck Medical University and patients had given informed consent (ID of ethical vote: 1167/2020).

### Laboratory Measurements

All analyses were conducted at the ISO 15189 accredited Central Institute of Clinical and Chemical Laboratory Diagnostics (Medical University of Innsbruck, Austria) according to the manufacturers’ procedures. Blood samples for detection of sex hormones were taken guideline conform till 11 a.m. within the first three days after hospital admission. A fully automated analyzer (Cobas 8000) by Roche Diagnostics GmbH (Mannheim, Germany) comprising an indirect potentiometric unit (ISE module), a chemistry unit (module 702), and an immunological unit (module e602) was used to determine the following parameters: creatinine (CREP2, enzymatic), aspartate transaminase (AST; ASTPM), alanine aminotransferase (ALT; ALTPM), alkaline phosphatase (ALP; ALP2), interleukin 6 (IL-6; Elecsys IL-6), cholesterol (CHOL2), high-density lipoprotein (HDL; HDLC4), low-density lipoprotein (LDL; LDLC3), triglycerides (TRIGL), estradiol (E_2_; Elecsys Estradiol III), procalcitonin (PCT; Elecsys BRAHMS PCT), C-reactive protein (CRP; CRP4), and ferritin (FERR4). Glycated hemoglobin (HbA1c) was measured by liquid chromatography on a TOSOH G8 instrument (Tosoh Corporation, Shiba, Minato-ku, Japan). Fibrinogen was determined on a Siemens analyzer (BCS-XP) using reagents from Siemens (Multifibren U). Androstenedione, dehydroepiandrosterone (as sulfate), luteinizing hormone (LH), follicle-stimulating hormone (FSH), and sexual hormone-binding globulin (SHBG) were quantified using reagents from Siemens on an IMMULITE ^®^ 2000 XP analyzer. Neopterin was measured using the ELISA from IBL International (Hamburg, Germany) on a Dynex DS2 automated ELISA system (Dynex Technologies, Chantilly, USA) and total testosterone (tT) was determined using high-pressure liquid chromatography hyphenated with tandem mass spectrometry *via* an in-house developed method. Free testosterone (fT) was measured *via* a CLIA assay obtained from IDS iSYS (Immunodiagnostic Systems GmbH, Frankfurt am Main, Germany) on an IDS-iSYS Multi-Discipline Automated System. All hematological parameters (thrombocytes, leucocytes, lymphocytes, hemoglobin, and hematocrit) were measured on a Sysmex automated hematology analyzer (XN series).

We calculated the luteinizing hormone to total testosterone (LH/tT) ratio to specify the hypothalamic-pituitary-gonadal axis (35) and the total testosterone to estradiol (tT/E_2_) ratio to reflect aromatase activity (36). Reference ranges for total testosterone (tT) levels were based on the guideline of the investigation, treatment and monitoring of functional hypogonadism in males by the European Academy of Andrology (EAA): serum total testosterone (tT) levels were classified to be reduced when tT levels ≤ 230 ng/dL, borderline when tT levels between 231 – 350 ng/dL and normal when tT levels > 350 ng/dL (37).

### Statistical Analyses

Parameters are reported as n (%) or medians (25th, 75th percentile) since the data were not normally distributed (Shapiro-Wilk test). To test for differences between men and women we used the Mann-Whitney-U test or Pearson chi-square tests. Kruskal-Wallis test was performed to test for significant differences between more than two groups. Analysis of the effect of risk factors on the probability of death or ICU admission during the in-hospital stay was performed with logistic regression analysis (not normally distributed parameters were logarithmized with the natural logarithm). All tests were two-tailed and p-values < 0.05 were regarded as statistically significant. Statistical analysis was performed using SPSS Statistics Version 27 (IBM Corporation, Armonk, NY, USA).

## Results

### Sex Differences in Baseline Characteristics

Within this retrospective analysis we investigated clinical, hormonal and inflammatory parameters in 230 men (61.0%) and 147 women (39.0%) with PCR-confirmed COVID-19 disease and a median age of 67 years in men and 70 years in women (p = 0.540). Upon initial hospital admission men presented with significantly lower serum cholesterol (121 *vs*. 141 mg/dL, p < 0.001), LDL (72 *vs*. 83 mg/dL, p = 0.001) and HDL levels (31 *vs*. 40 mg/dL, p < 0.001) as well as significantly higher immune activation markers, namely C-reactive protein (CRP; 6.01 mg/dL *vs*. 3.70 mg/dL, p < 0.001), interleukin 6 (IL-6; 44.5 *vs*. 24.0 ng/L, p < 0.001), procalcitonin (PCT; 0.13 *vs*. 0.07 ng/mL, p < 0.001), neopterin (51.2 *vs*. 41.3 nmol/L, p = 0.002), fibrinogen (511 *vs*. 439 G/L, p = 0.002) and ferritin levels (662 *vs*. 265 μg/L, p < 0.001) as well as higher leukocytes counts (5.80 *vs*. 5.00 G/L, p = 0.003) compared to women. The prevalence of cardiovascular disease (53.2 % *vs*. 49.0 %, p = 0.007) and diabetes mellitus (35.7 % *vs*. 25.9 %, p = 0.046) was also significantly higher in men than women. Finally, men were at higher risk to die during hospital stay (16.2 % *vs*. 6.8 %, p = 0.008) with significantly longer hospitalizations compared to women (11 *vs*. 8 days, p = 0.002). We will focus on sex hormones in SARS-CoV-2 infected men in the following analysis. Baseline characteristics for men upon hospital admission are depicted in **Table 1**.

**Table 1:** Baseline characteristics of men with corresponding reference ranges.

	All men	Men with available sex hormones	Reference
	n = 230	n = 155	
	Median (IQR) or n (%)	Median (IQR) or n (%)	
Clinical characteristics			
age [years]	67 (54 – 78)	66 (53 – 78)	
BMI [kg/m^2^]	26.4 (24.1 – 29.4)	26.4 (24.2 – 29.3)	
Temperature [°C]	37.8 (36.8 – 38.6)	37.9 (37.0 – 38.7)	
SpO_2_ [%]	91.5 (88.0 – 94.0)	92.0 (88.0 – 94.0)	
O_2_ requirement [L]	2.0 (0.0 – 4.0)	2.0 (0.0 – 4.0)	
WHO score	4 (4 – 5)	4 (4 – 5)	
Hospitalization, days^‡^	11 (7 – 16)	9 (6 – 13)	
ICU admission^#^	20.5%	20.5%	
Death during hospital stay	16.1%	12.9%	
Duration symptoms till hospitalization, days	6 (3 – 10)	9 (6 – 12)	
Comorbidities and risk factors			
Cardiovascular disease	63.2%	61.0%	
Arterial hypertension	46.5%	46.8%	
Diabetes Mellitus	35.7%	27.7%	
Chronic Kidney Disease	13.5%	15.5%	
Malignancies	13.6%	13.0%	
COPD	8.3%	6.5%	
Bronchial asthma	5.2%	6.5%	
Nicotine abuse	20.5%	21.9%	
Concomitant Medication			
Therapy for BPH	10.1%	9.7%	
Lipid lowering drugs	29.1%	29.0%	
General laboratory parameters			
eGFR [mL/min/1.73m^2^]	77.66 (52.51 – 95.17)	76.54 (56.05 – 95.17)	>60
AST [U/L]	37 (28 – 59)	36 (28 – 59)	10 – 50
ALT [U/L]	28 (19 – 46)	27 (18 – 46)	10 – 50
ALP [U/L]	65 (51 – 86)	67 (54 – 80)	40 – 129
HbA1c [%]	6.2 (5.7 – 6.8)	6.1 (5.7 – 6.8)	4.8 – 5.7
Thrombocytes [G/L]	182 (140 – 243)	178 (137 – 237)	150 – 380
Hemoglobin [g/L]	137 (124 – 150)	136 (124 – 149)	130 – 177
Hematocrit [L/L]	0.396 (0.358 – 0.437)	0.396 (0.359 – 0.429)	0.400 – 0.520
Cholesterol [mg/dL]	121 (97 – 148)	122 (97 – 147)	130 – 200
LDL [mg/dL]	72 (50 – 95)	72 (50 – 94)	0 – 116
HDL [mg/dL]	31 (24 – 39)	32 (24 – 39)	>40
Triglycerides [mg/dL]	104 (84 – 135)	106 (85 – 138)	40 – 150
Inflammatory biomarkers			
CRP [mg/dL]	6.01 (2.09 – 12.29)	5.25 (1.57 – 11.10)	0.00 – 0.50
IL-6 [ng/L]	44.5 (17.3 – 91.4)	38.3 (14.4 – 81.1)	0.00 – 0.50
IL-10 [pg/mL]	5.60 (2.90 – 10.20)	5.60 (2.90 – 10.20)	0.00 – 3.50
PCT [ng/mL]	0.13 (0.07 – 0.33)	0.11 (0.07 – 0.32)	0.00 – 0.50
Neopterin [nmol/L]	51.2 (31.5 – 81.3)	50.3 (31.4 – 78.2)	0.0 – 10.0
Fibrinogen [G/L]	511 (398 – 592)	498 (392 – 586)	210 – 400
Ferritin [μg/L]	662 (348 – 1.371)	535 (313 – 1140)	30 – 400
Leukocytes [G/L]	5.80 (4.40 – 8.10)	5.70 (4.30 – 7.50)	4.0 – 10.0
Lymphocytes absolute [G/L]	0.97 (0.65 – 1.39)	1.01 (0.71 – 1.40)	0.80 – 4.00
Hormones			
Total testosterone [ng/dL]	148 (70 – 270)	148 (70 – 270)	>350
Free testosterone [ng/L]	4.01 (2.47 – 5.87)	4.01 (2.47 – 5.87)	4.81 – 22.42
DHEA [mg/L]	0.70 (0.33 – 1.09)	0.70 (0.33 – 1.09)	1.29 – 4.09
Androstenedione [μg/L]	1.3 (0.6 – 1.9)	1.3 (0.6 – 1.9)	0.6 – 3.1
Estradiol [ng/L]	28 (24 – 38)	28 (24 – 38)	11 – 43
SHBG [nmol/L]	31.0 (21.2 – 41.5)	31.0 (21.2 – 41.5)	10.0 – 57.0
LH [U/L]	6.0 (4.0 – 9.4)	6.0 (4.0 – 9.4)	0.8 – 7.6
FSH [U/L]	5.3 (3.0 – 11.1)	5.3 (3.0 – 11.1)	1.6 – 20.4
TSH [mU/L]	1.00 (0.68 – 1.68)	1.02 (0.70 – 1.65)	0.35 – 3.50
FT4 [pmol/L]	15.4 (13.4 – 17.7)	15.1 (13.3 – 17.4)	10.3 – 21.9
FT3 [pmol/L]	3.23 (2.60 – 3.95)	3.26 (2.72 – 3.99)	2.50 – 6.70

^‡^without patients who died during hospital stay, ^#^only patients over the age of 80 (n = 127).

IQR, interquartile range; BMI, body mass index; SpO2, peripheral capillary oxygen saturation; O2, oxygen; WHO, World Health Organization; ICU, intensive care unit; COPD, Chronic obstructive pulmonary disease; BPH, benign prostatic hyperplasia; eGFR, estimated glomerular filtration rate; AST, aspartate aminotransferase; ALT, alanine aminotransferase; ALP, alkaline phosphatase; CRP, C-reactive protein; IL-6, interleukin 6; IL-10, interleukin 10; PCT, procalcitonin; HbA1c, glycated hemoglobin; HDL, high density lipoprotein; LDL, low density lipoprotein; TSH, thyroid stimulating hormone; FT4, free thyroxine; FT3, free triiodothyronine; LH, luteinizing hormone; FSH, follicle-stimulating hormone; DHEA, dehydroepiandrosterone; SHBG, sexual hormone binding globulin.

### Baseline Sex Hormones and Lipids in Men With COVID-19

Sex hormones were available from 267 patients (155 men, 112 women) in the first three days after hospital admission. Most hospitalized men with available sex hormones upon initial hospital admission (n = 155) presented with reduced total testosterone (tT) levels ≤ 230 ng/dL (n = 107, 69.0 %), while 22 men (14.2 %) presented with borderline tT levels between 231 – 350 ng/dL and 24 men (15.5 %) with normal tT levels > 350 ng/dL. When differentiating men according to their age, men over the age of 60 had a significantly higher prevalence of reduced tT levels (81.5% *vs*. 52.5 %) and a lower prevalence of borderline tT levels (9.8 % *vs*. 21.3 %) when compared to men under the age of 60 (p < 0.001). Irrespective of the median age of 67 years, only 15 men out of 155 (9.7 %) were on pharmacological therapy for their benign prostate enlargement with a 5-alpha-reductase inhibitor, generally accepted to not influence the tT concentrations; indeed, these men showed median tT levels of 191 ng/dL (65 – 419 ng/dL) and median free testosterone (fT) levels of 3.94 ng/L (2.05 – 5.65 ng/L) as compared to men without 5-alpha reductase inhibitor therapy with tT levels of 148 ng/dL (70 – 262 ng/dL, p = 0.494) and fT levels of 4.19 ng/L (2.49 – 5.89 ng/L, p = 0.510). Interestingly, although tT levels were rather low in the majority of hospitalized men, levels of the gonadotropins luteinizing hormone (LH) and follicle-stimulating hormone (FSH) as well as the steroid hormone estradiol (E_2_) were within the upper normal range (**Table 1**). However, when differentiating men again according to their age, men over the age of 60 presented with slightly elevated LH levels (7.6 U/L [4.8 – 12.0]) which were also significantly higher compared to men under the age of 60 (4.8 [3.5 – 6.1], p < 0.001). Conversely, tT levels were significantly lower in men over the age of 60 compared to men under the age of 60 (130 ng/dL [50 – 198] *vs*. 219 ng/dL [120 – 359], p < 0.001),

Cardiovascular disease, arterial hypertension, diabetes mellitus and hypercholesterinemia were frequently encountered in men within our study. Interestingly, serum low-density lipoprotein (LDL) and high-density lipoprotein (HDL) were quite low even in men without *vs*. with lipid-lowering therapy (LDL: 81 mg/dL *vs*. 57 mg/dL; HDL: 32 mg/dL *vs*. 30 mg/dL). Low serum cholesterol, LDL and HDL levels were associated with older age, higher immune activation, WHO-score and temperature and lower SpO_2_ with concomitant higher O_2_ requirements (**Table 2**). Moreover, low tT levels as well as a lower tT/E_2_ and higher LH/tT ratio were associated with lower cholesterol, LDL and HDL levels (**Table 3**).

**Table 2:** Correlations of lipoproteins and thyroid hormones with clinical characteristics and laboratory parameters in men.*

	Cholesterol	LDL	HDL	Triglycerides	TSH	FT4	FT3
	[mg/dL]	[mg/dL]	[mg/dL]	[mg/dL]	[mU/L]	[pmol/L]	[pmol/L]
Clinical characteristics							
Age	- 0.124	**- 0.194**	**0.159**	- 0.132	- 0.043	- 0.062	**- 0.473**
[years]	p = 0.128	**p = 0.016**	**p = 0.050**	p = 0.108	p = 0.595	p = 0.456	**p < 0.001**
BMI	- 0.126	- 0.063	**- 0.248**	0.194	0.050	0.088	0.087
[kg/m^2^]	p = 0.142	p = 0.466	**p = 0.003**	p = 0.023	p = 0.554	p = 0.314	p = 0.324
Temp.	**- 0.189**	**- 0.186**	- 0.124	- 0.016	- 0.094	- 0.059	**- 0.192**
[°C]	**p = 0.021**	**p = 0.023**	p = 0.130	p = 0.845	p = 0.250	p = 0.482	**p = 0.022**
SpO_2_	**0.171**	**0.202**	**0.214**	- 0.119	0.046	0.074	**0.315**
[%]	**p = 0.038**	**p = 0.013**	**p = 0.009**	p = 0.151	p = 0.571	p = 0.380	**p < 0.001**
O_2_ req.	**- 0.275**	**- 0.274**	**- 0.360**	0.184	- 0.076	- 0.131	**- 0.358**
[L]	**p < 0.001**	**p < 0.001**	**p < 0.001**	p = 0.025	p = 0.355	p = 0.119	**p < 0.001**
WHO	**- 0.264**	**- 0.348**	**- 0.263**	0.155	- 0.151	- 0.082	**- 0.401**
score	**p = 0.001**	**p < 0.001**	**p = 0.001**	p = 0.060	p = 0.062	p = 0.329	**p < 0.001**
Hosp.	**- 0.218**	**- 0.219**	- 0.047	- 0.037	- 0.029	- 0.007	**- 0.256**
[days] ^‡^	**p = 0.013**	**p = 0.013**	p = 0.598	p = 0.676	p = 0.744	p = 0.943	**p = 0.005**
Inflammatory biomarkers							
CRP	**- 0.347**	**- 0.382**	**- 0.369**	0.136	- 0.129	- 0.142	**- 0.452**
[mg/dL]	**p < 0.001**	**p < 0.001**	**p < 0.001**	p = 0.096	p = 0.112	p = 0.088	**p < 0.001**
IL-6	**- 0.384**	**- 0.384**	**- 0.246**	- 0.044	- 0.054	- 0.136	**- 0.427**
[ng/L]	**p < 0.001**	**p < 0.001**	**p = 0.002**	p = 0.589	p = 0.508	p = 0.103	**p < 0.001**
IL-10	**- 0.213**	**- 0.228**	- 0.025	0.063	0.057	- 0.006	**- 0.224**
[pg/mL]	**p = 0.030**	**p = 0.019**	p = 0.802	p = 0.522	p = 0.562	p = 0.953	**p = 0.022**
PCT	**- 0.346**	**- 0.402**	**- 0.273**	0.109	- 0.076	**- 0.270**	**- 0.470**
[ng/mL]	**p < 0.001**	**p < 0.001**	**p < 0.001**	p = 0.184	p = 0.350	**p = 0.001**	**p < 0.001**
Neopterin	**- 0.242**	**- 0.322**	- 0.086	0.039	- 0.079	**- 0.215**	**- 0.493**
[nmol/L]	**p = 0.003**	**p < 0.001**	p = 0.290	p = 0.634	p = 0.329	**p = 0.009**	**p < 0.001**
Fibrinogen	**- 0.224**	**- 0.221**	**- 0.304**	0.145	- 0.027	- 0.057	**- 0.258**
[G/L]	**p = 0.007**	**p = 0.007**	**p < 0.001**	p = 0.082	p = 0.742	p = 0.504	**p = 0.002**
Ferritin	**- 0.199**	**- 0.259**	**- 0.270**	**0.238**	**- 0.202**	- 0.065	**- 0.236**
[μg/L]	**p = 0.014**	**p = 0.001**	**p < 0.001**	**p = 0.003**	**p = 0.012**	p = 0.439	**p = 0.004**
Leukocytes	0.025	- 0.002	- 0.071	0.098	- 0.116	0.095	- 0.022
[G/L]	p = 0.764	p = 0.979	p = 0.388	p = 0.235	p = 0.152	p = 0.256	p = 0.793
Lymph.	**0.216**	**0.223**	0.124	0.016	**0.177**	0.149	**0.333**
abs. [G/L]	**p = 0.008**	**p = 0.006**	p = 0.129	p = 0.844	**p = 0.028**	p = 0.075	**p < 0.001**

*Spearman-rank correlation coefficient with according p-Value, ^‡^without patients who died during hospital stay. Statistically significant correlations are marked in bold. LDL, low density lipoprotein; HDL, high density lipoprotein; TSH, thyroid stimulating hormone; FT4, free thyroxine; FT3, free triiodothyronine; tT, total testosterone; fT, free testosterone; DHEA, dehydroepiandrosterone; ASD, Androstenedione; E_2_, estradiol; SHBG, sexual hormone binding globulin; LH = luteinizing hormone; FSH = follicle-stimulating hormone; BMI, body mass index; Temp., temperature; SpO2, peripheral capillary oxygen saturation; O_2_ req., oxygen requirement; WHO, World Health Organization; Hosp., hospitalization duration; CRP, C-reactive protein; IL-6, interleukin 6; IL-10, interleukin 10; PCT, procalcitonin; Lymph. abs., lymphocytes absolute.

**Table 3:** Correlations of sex hormones with clinical characteristics and laboratory parameters in men.*

	tT	fT	DHEA	ASD	E_2_	SHBG	LH	FSH	LH/tT	tT/E_2_
	[ng/dL]	[ng/L]	[mg/dL]	[μg/L]	[ng/L]	[nmol/L]	[U/L]	[U/L]	ratio	ratio
Clinical characteristics										
Age	**- 0.361**	**- 0.417**	**- 0.568**	0.116	0.095	**0.455**	**0.434**	**0.402**	**0.485**	**- 0.379**
[years]	**p < 0.001**	**p < 0.001**	**p < 0.001**	p = 0.154	p = 0.243	**p <0.001**	**p < 0.001**	**p < 0.001**	**p < 0.001**	**p < 0.001**
BMI	- 0.135	- 0.046	0.090	- 0.082	0.042	**- 0.279**	- 0.064	0.018	0.063	- 0.153
[kg/m^2^]	p = 0.114	p = 0.589	p = 0.291	p = 0.335	p = 0.624	**p = 0.002**	p = 0.473	p = 0.842	p = 0.482	p = 0.072
Temp.	**- 0.245**	**0.237**	0.002	0.070	- 0.012	- 0.115	0.067	0.067	**0.213**	**- 0.241**
[°C]	**p = 0.002**	**p = 0.003**	p = 0.978	p = 0.394	p = 0.883	p = 0.191	p = 0.436	p = 0.434	**p = 0.012**	**p = 0.003**
SpO_2_	**0.500**	**0.237**	**0.190**	- 0.074	**- 0.172**	0.065	- 0.160	- 0.006	**- 0.472**	**0.530**
[%]	**p < 0.001**	**p = 0.003**	**p = 0.020**	p = 0.365	**p = 0.035**	p = 0.456	p = 0.062	p = 0.946	**p < 0.001**	**p < 0.001**
O_2_ req.	**- 0.512**	**- 0.197**	**- 0.187**	- 0.055	0.157	**- 0.208**	0.082	- 0.077	**0.454**	**- 0.557**
[L]	**p < 0.001**	**p = 0.015**	**p = 0.022**	p = 0.503	p = 0.055	**p = 0.017**	p = 0.339	p = 0.370	**p < 0.001**	**p < 0.001**
WHO	**- 0.552**	**- 0.384**	**- 0.273**	0.048	0.093	- 0.005	0.079	- 0.032	**0.488**	**- 0.569**
score	**p < 0.001**	**p < 0.001**	**p = 0.001**	p = 0.561	p = 0.256	ü = 0.953	p = 0.357	p = 0.708	**p < 0.001**	**p < 0.001**
Hosp.	**- 0.256**	**- 0.305**	**- 0.226**	0.004	0.013	**0.232**	**0.146**	**0.087**	**0.267**	**- 0.262**
[days] ^‡^	**p = 0.003**	**p < 0.001**	**p = 0.010**	p = 0.966	p = 0.887	**p = 0.014**	**p = 0.119**	**p = 0.354**	**p = 0.004**	**p = 0.003**
Inflammatory biomarkers										
CRP	**- 0.567**	**- 0.252**	- 0.091	0.076	**0.163**	- 0.156	0.017	**- 0.178**	**0.435**	**- 0.596**
[mg/dL]	**p < 0.001**	**p = 0.002**	p = 0.262	p = 0.349	**p = 0.045**	p = 0.072	p = 0.846	**p = 0.036**	**p < 0.001**	**p < 0.001**
IL-6	**- 0.563**	**- 0.364**	**- 0.237**	**0.198**	**0.165**	- 0.035	0.126	0.004	**0.536**	**- 0.595**
[ng/L]	**p < 0.001**	**p < 0.001**	**p = 0.003**	**p = 0.014**	**p = 0.041**	p = 0.688	p = 0.139	p = 0.959	**p < 0.001**	**p < 0.001**
IL-10	**- 0.376**	**- 0.332**	**- 0.206**	**- 0.062**	- 0.123	**- 0.204**	0.050	- 0.002	**0.362**	**- 0.339**
[pg/mL]	**p < 0.001**	**p < 0.001**	**p = 0.034**	**p = 0.531**	p = 0.210	**p = 0.047**	p = 0.621	p = 0.983	**p < 0.001**	**p < 0.001**
PCT	**- 0.542**	**- 0.233**	- 0.133	0.152	**0.257**	- 0.020	0.056	- 0.054	**0.445**	**- 0.616**
[ng/mL]	**p < 0.001**	**p = 0.004**	p = 0.102	p = 0.061	**p = 0.001**	p = 0.819	p = 0.514	p = 0.531	**p < 0.001**	**p < 0.001**
Neopterin	**- 0.392**	**- 0.319**	**- 0.375**	0.099	0.114	0.167	**0.347**	**0.252**	**0.532**	**- 0.439**
[nmol/L]	**p < 0.001**	**< 0.001**	**p < 0.001**	ü = 0.223	p = 0.159	p = 0.054	**p < 0.001**	**p = 0.003**	**p < 0.001**	**p < 0.001**
Fibrinogen	**- 0.420**	- 0.139	**0.038**	0.059	0.132	**- 0.231**	- 0.003	**- 0.212**	**0.305**	**- 0.540**
[G/L]	**p < 0.001**	p = 0.090	**p = 0.643**	p = 0.473	p = 0.110	**p = 0.008**	p = 0.973	**p = 0.013**	**p < 0.001**	**p < 0.001**
Ferritin	**- 0.272**	- 0.001	0.070	0.022	**0.326**	- 0.012	- 0.033	**- 0.178**	**0.194**	**- 0.387**
[μg/L]	**p = 0.001**	p = 0.988	p = 0.391	p = 0.785	**p < 0.001**	p = 0.888	p = 0.697	**p = 0.036**	**p = 0.023**	**p < 0.001**
Leukocytes	**- 0.166**	0.006	0.000	**0.159**	0.146	- 0.025	- 0.054	- 0.152	0.164	**- 0.204**
[G/L]	**p = 0.040**	p = 0.940	p = 1.000	**p = 0.049**	p = 0.071	p = 0.772	p = 0.528	p = 0.073	p = 0.055	**p = 0.011**
Lymph.	**0.357**	**0.302**	**0.169**	0.101	- 0.079	- 0.027	- 0.137	0.019	**- 0.299**	**0.358**
abs. [G/L]	**p < 0.001**	**p < 0.001**	**p = 0.037**	p = 0.215	p = 0.331	p = 0.760	p = 0.109	p = 0.827	**p < 0.001**	**p < 0.001**
Lipoproteins and thyroid hormones										
Cholesterol	**0.407**	0.147	0.142	0.127	- 0.107	0.124	- 0.112	0.056	**- 0.361**	**0.447**
[mg/dL]	**p < 0.001**	p = 0.073	p = 0.083	p = 0.123	p = 0.196	p = 0.159	p = 0.195	p = 0.516	**p < 0.001**	**p < 0.001**
LDL	**0.430**	**0.183**	**0.197**	0.063	- 0.081	0.061	- 0.160	0.013	**- 0.407**	**0.461**
[mg/dL]	**p < 0.001**	**p = 0.025**	**p = 0.015**	p = 0.446	p = 0.327	p = 0.491	p = 0.061	p = 0.884	**p < 0.001**	**p < 0.001**
HDL	**0.345**	0.019	0.068	0.101	0.001	**0.296**	0.086	**0.188**	**- 0.232**	**0.346**
[mg/dL]	**p < 0.001**	p = 0.813	p = 0.409	p = 0.221	p = 0.986	**p < 0.001**	p = 0.316	**p = 0.028**	**p = 0.007**	**p < 0.001**
Triglycerides	- 0.082	0.072	- 0.050	0.035	0.012	**- 0.224**	- 0.024	- 0.037	0.114	- 0.079
[mg/dL]	p = 0.323	p = 0.386	p = 0.549	p = 0.677	p = 0.890	**p = 0.010**	p = 0.782	p = 0.669	p = 0.187	p = 0.341
TSH	0.015	- 0.005	- 0.015	0.097	- 0.067	**- 0.198**	- 0.042	0.047	- 0.039	0.038
[mU/L]	p = 0.858	p = 0.955	p = 0.851	p = 0.235	p = 0.410	**p = 0.022**	p = 0.623	p = 0.581	p = 0.652	p = 0.640
FT4	**0.171**	0.126	0.049	- 0.034	- 0.081	0.067	- 0.030	- 0.078	- 0.111	**0.169**
[pmol/L]	**p = 0.041**	p = 0.130	p = 0.559	p = 0.688	p = 0.337	p = 0.450	p = 0.727	p = 0.367	p = 0.202	**p = 0.043**
FT3	**0.505**	**0.426**	**0.381**	- 0.074	- 0.019	**- 0.173**	- 0.144	- 0.139	**- 0.452**	**0.486**
[pmol/L]	**p < 0.001**	**p < 0.001**	**p < 0.001**	p = 0.376	p = 0.823	**p = 0.049**	p = 0.096	p = 0.107	**p < 0.001**	**p < 0.001**

*Spearman-rank correlation coefficient with according p-Value, ^‡^without patients who died during hospital stay. Statistically significant correlations are marked in bold.

tT, total testosterone; fT, free testosterone; DHEA, dehydroepiandrosterone; ASD, Androstenedione; E_2_, estradiol; SHBG, sexual hormone binding globulin; LH = luteinizing hormone; FSH = follicle-stimulating hormone; BMI, body mass index; Temp., temperature; SpO2, peripheral capillary oxygen saturation; O_2_ req., oxygen requirement; WHO, World Health Organization; Hosp., hospitalization duration; CRP, C-reactive protein; IL-6, interleukin 6; IL-10, interleukin 10; PCT, procalcitonin; Lymph. abs., lymphocytes absolute; LDL, low density lipoprotein; HDL, high density lipoprotein; TSH, thyroid stimulating hormone; FT4, free thyroxine; FT3, free triiodothyronine.

### Sex Hormones, Disease Severity and Immune Activation

In men (n = 155) lower tT and fT levels upon hospital admission were correlated with older age, higher WHO score and temperature, as well as lower SpO_2_ with concomitant higher O_2_ requirement and longer hospital stay (**Table 3**). Also, lower dehydroepiandrosterone (DHEA) levels were significantly correlated with an older age, higher WHO score and lower SpO_2_, again with higher O_2_ requirement and longer hospital stay. SHBG was positively correlated with age and hospitalization duration and negatively with BMI and O_2_ requirement. Androstenedione (ASD), E_2_, LH and FSH were not related to disease severity. Finally, a higher LH/tT and a lower tT/E_2_ ratio were associated with an older age, higher WHO score and temperature as well as lower SpO_2_ with accordingly higher O_2_ requirements and longer hospital stay (**Table 3**).

When analyzing relations of sex hormones with markers of immune activation, tT and fT levels negatively correlated with CRP, IL-6, IL-10, neopterin, PCT, fibrinogen and ferritin levels as well as with leukocyte counts and positively correlated with absolute lymphocyte numbers. Also, other sex hormones correlated widely with different biomarkers of immune activation depicted in **Table 3**. Interestingly, a higher LH/tT and lower tT/E_2_ ratio was associated with higher CRP, IL-6, IL-10, neopterin, PCT, fibrinogen and ferritin levels as well as lower absolute lymphocyte counts. (**Table 3**)

### Testosterone and Estradiol Levels Predict the Outcome in Men With COVID-19

**Figure 1:**
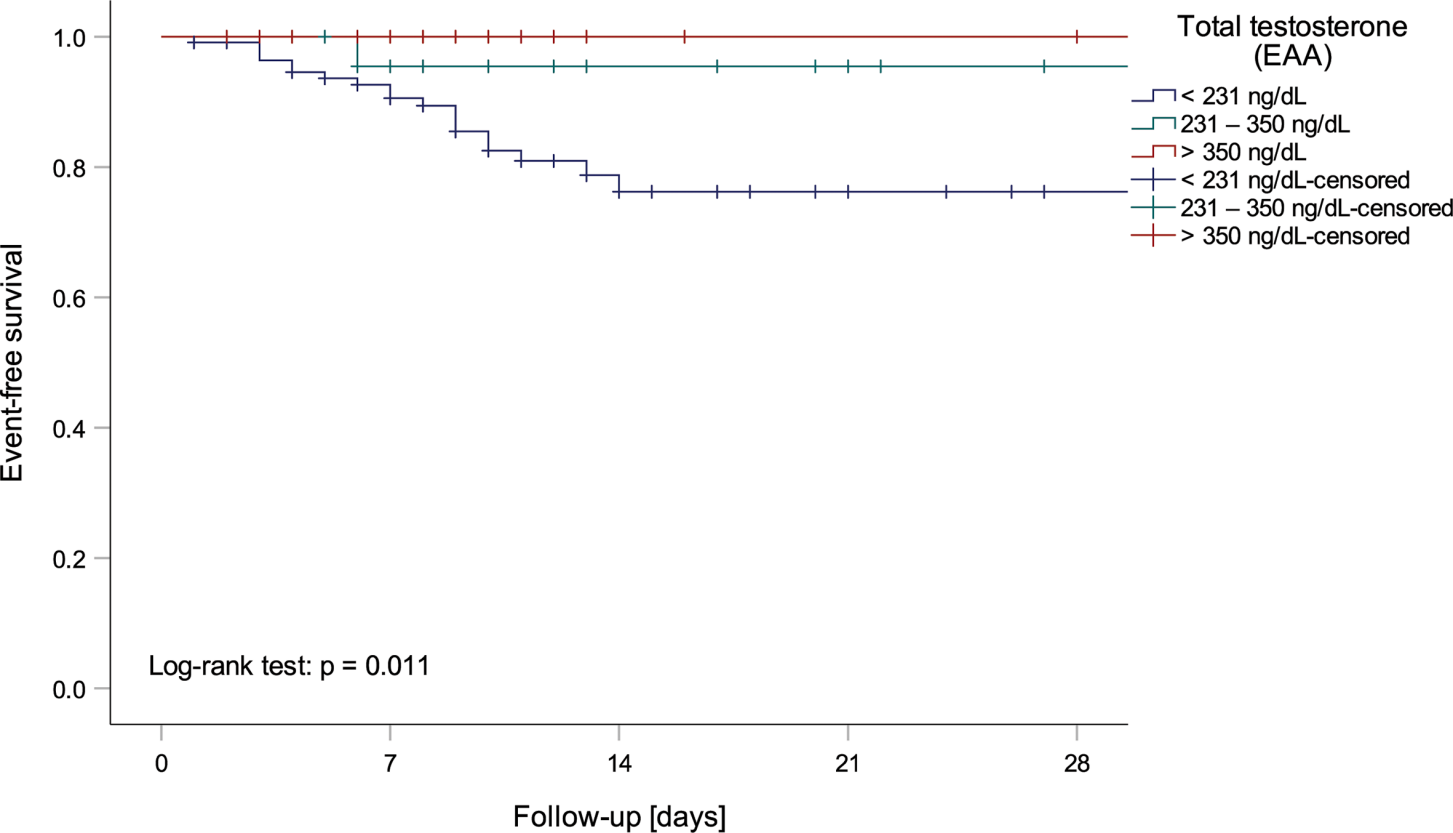
Kaplan-Meier curve depicting mortality of men within testosterone normality ranges by the European Academy of Andrology (EAA) (37).

Univariate logistic regression analysis showed that lower tT, fT and DHEA levels, but higher ASD and E_2_ levels, as well as a lower tT/E_2_ and higher LH/tT ratio, were associated with an increased risk to die during hospital stay (**Table 4**). Men with tT levels < 100 ng/dL (n = 52) had a more than eighteen-fold higher risk to die during hospital stay when compared to men with tT levels > 230 ng/dL (n = 46; OR 18.243 [95%CI 2.301 – 144.639], p = 0.006, **Figure 1**). Actually, 19 out of 20 men who died had reduced tT levels ≤ 230 ng/dL; yet one man who died had a tT level of 244 ng/dL.

**Table 4:** Logistic regression analysis of sex hormones and the risk do die or admission to ICU during hospital stay in men.

	Univariate Model				Multivariate Model *			
	Wald	HR	95% CI	p-Value	Wald	HR	95% CI	p-Value
Risk to die during hospital stay								
tT [ng/dL] _Ln	**16.184**	**0.340**	**0.201 - 0.575**	**<0.001**	2.095	0.574	0.270 - 1.217	0.148
fT [ng/L] _Ln	**5.145**	**0.512**	**0.287 - 0.913**	**0.023**	0.073	0.887	0.372 - 2.117	0.787
DHEA [mg/L] _Ln	**5.786**	**0.365**	**0.160 - 0.830**	**0.016**	0.038	1.133	0.323 - 3.970	0.845
ASD [μg/L] _Ln	**4.584**	**2.076**	**1.064 - 4.051**	**0.032**	0.004	1.028	0.439 - 2.406	0.949
E_2_, [ng/L] _Ln	**11.612**	**5.243**	**2.022 - 13.598**	**<0.001**	**4.807**	**6.554**	**1.221 - 35.187**	**0.028**
SHBG [nmol/L] _Ln	1.580	1.841	0.711 - 4.768	0.209	0.521	0.527	0.093 - 3.000	0.470
LH [U/L] _Ln	0.382	1.235	0.633 - 2.411	0.536	1.220	0.601	0.243 - 1.483	0.269
FSH [U/L] _Ln	0.155	1.108	0.665 - 1.845	0.694	2.456	0.519	0.229 - 1.179	0.117
LH/tT ratio _Ln	**11.334**	**2.045**	**1.348 - 3.100**	**<0.001**	0.088	1.093	0.608 - 1.965	0.767
tT/E_2_ ratio _Ln	**21.680**	**0.313**	**0.192 - 0.510**	**<0.001**	**4.616**	**0.440**	**0.208 - 0.931**	**0.032**
Risk for ICU admission during hospital stay ^†,‡^								
tT [ng/dL] _Ln	**18.978**	**0.247**	**0.132 - 0.463**	**<0.001**	**9.398**	**0.314**	**0.150 - 0.658**	**0.002**
fT [ng/L] _Ln	**15.933**	**0.189**	**0.083 - 0.428**	**<0.001**	**7.376**	**0.269**	**0.105 - 0.694**	**0.007**
DHEA [mg/L] _Ln	2.279	0.601	0.310 - 1.164	0.131	0.000	0.993	0.428 - 2.304	0.987
ASD [μg/L] _Ln	2.054	0.642	0.351 - 1.177	0.152	1.974	0.599	0.293 - 1.224	0.160
E_2_, [ng/L] _Ln	1.274	1.932	0.616 - 6.062	0.259	0.186	1.338	0.356 - 5.028	0.667
SHBG [nmol/L] _Ln	0.118	0.849	0.333 - 2.166	0.732	0.134	0.794	0.231 - 2.729	0.715
LH [U/L] _Ln	0.026	1.064	0.499 - 2.269	0.871	0.072	0.883	0.356 - 2.189	0.788
FSH [U/L] _Ln	3.119	0.588	0.326 - 1.060	0.077	**3.984**	**0.486**	**0.240 - 0.987**	**0.046**
LH/tT ratio _Ln	**12.106**	**2.447**	**1.478 - 4.052**	**<0.001**	**6.260**	**2.242**	**1.191 - 4.221**	**0.012**
tT/E_2_ ratio _Ln	**19.583**	**0.280**	**0.159 - 0.492**	**<0.001**	**9.043**	**0.348**	**0.175 - 0.692**	**0.003**

*adjusted for age, BMI, LDL, the time of symptom onset and concomitant antiandrogen therapy, ^†^only patients under the age of 80 (n = 127), ^‡^without patients who died during hospital stay. Statistically significant results are marked in bold.

tT, total testosterone; fT, free testosterone; DHEA, dehydroepiandrosterone; ASD, Androstenedione; E_2_, estradiol; SHBG, sexual hormone binding globulin; LH, luteinizing hormone; FSH, follicle-stimulating hormone; BMI, body mass index; LDL, low density lipoprotein.

Moreover, men with a tT/E_2_ ratio < 3.30 (n = 52) had a more than 22-fold higher mortality risk when compared to men with a tT/E_2_ ratio > 7.30 (n = 51; OR 22.222 [95%CI 2.818 – 175.265], p = 0.003), while those with a LH/tT ratio > 6.95 (n = 46) had a more than 15-fold higher mortality risk when compared to men with a LH/tT ratio < 2.85 (n = 45; OR 15.529 [95%CI 1.924 – 125.367], p = 0.010). However, in multivariate logistic regression analysis adjusted for age, BMI, LDL, and the time of symptom onset only E_2_ and the tT/E_2_ ratio were significantly predicting mortality (**Table 4**).

Since men over the age of 80 were less probably transferred to the ICU because of their age, we only included men below the age of 80 (n = 127) in the following analyses. Increased risk for ICU admission during hospital stay was found in men with lower baseline tT or fT levels as well as in those with a lower tT/E_2_ ratio and higher LH/tT ratio. These findings were independent of age, BMI, LDL and the time of symptom onset in multivariate Cox regression analysis (**Table 4**). All 26 men who subsequently needed be transferred to the ICU had median tT baseline levels of only 80 ng/dL (range: 41 – 122 ng/dL), thus were hypogonadal per definition. Further subdivision in tertiles showed that men with tT levels < 100 ng/dL (n = 37) had a 12-fold higher risk for ICU admission compared to men with tT levels ≥ 100 ng/dL (n = 88; OR 12.214 [95%CI 4.467 – 33.398], p < 0.001). Additionally, men with a tT/E_2_ ratio < 3.30 (n = 37) had a more than 39-fold higher risk for ICU admission compared to men with a tT/E_2_ > 7.30 (n = 47; OR 39.100 [95%CI 4.865 – 314.225], p < 0.001), while men with a LH/tT ratio > 6.95 (n = 32) had a 24-fold higher risk for ICU admission compared to men with a LH/tT ratio < 2.85 (n = 41; OR 24.000 [95%CI 2.911 – 197.845], p = 0.003).

## Discussion

In the present study we found that low serum total and free testosterone levels upon hospital admission are associated with disease severity indicated by lower oxygen saturation, higher WHO score and increased risk for ICU admission or death during the hospital stay in men with SARS-CoV-2 infection. These data are in line with recent published small cohort studies suggesting that low testosterone levels predict clinical adverse outcome (38, 39). Our results strongly suggest that SARS-CoV-2 infected men with the need for hospitalization present with distinct low testosterone levels that are further not compensated by hypothalamic-hypophyseal feedbacks especially in younger men, since LH levels were within the normal range. Testosterone itself was shown to be associated with a reduced cytokine response following cellular immune activation in men but not in women (40). This might be due to already rather low testosterone levels in women who are therefore not further strongly affected by inflammation (41). In addition, androgen receptor expression in male immune cells is more distinctive than in female ones (18, 42). Thus testosterone is suggested to be partly responsible for the lower prevalence of autoimmune disease but also for the higher incidence of cancer in men compared to women (43). Interestingly, it was also shown that androgens can reduce lung pathology in influenza-infected mice (44) suggesting that testosterone prevents inappropriate overwhelming immune activation following infection (45). In the case of low testosterone levels, such suppressive mechanism may be reduced, which might result in an imbalance between immunosuppressive and pro-inflammatory regulating mechanism in men (46). It’s generally assumed that this can lead to enhanced pro-inflammatory cytokine response following a SARS-CoV-2 infection, also known as a cytokine storm. Therefore, a reduced innate immune response toward viral infections in men compared to women results in a higher and longer persisting viral load (47, 48) with a subsequently more pronounced cellular immune response (49). Such situation is mainly present in men with low serum levels of the immunosuppressive acting testosterone. Accordingly, serum testosterone levels were negatively correlated with all investigated inflammatory biomarkers including IL-6, IL-10, neopterin (Th1 immune response), PCT, CRP, fibrinogen and ferritin which all were associated with a poorer clinical course and higher risk for ICU admission or death in SARS-CoV-2 infected patients (10, 48, 50, 51). Vice versa, it was shown in previous studies, that the testosterone production in Leydig cells is also downregulated by immune activation itself (52). The findings of our study, that rather low than high testosterone levels predict mortality in men, contrast with recent findings showing that androgen stimulates the expression of the ACE2 coreceptor TMPRSS2 (53), which further provides access of SARS-CoV-2 to cells (54), suggesting that higher testosterone levels with consequently higher receptor density provides more docking sites for SARS-CoV-2. However, high mortality in COVID-19 is suggested to be caused by hyperinflammation and not actually by higher viral loads (55). Thus, results of our study suggest that low levels of testosterone might be even more immunosuppressive than high testosterone levels with consequently higher SARS-CoV-2 receptor expression.

However, other risk factors for severe COVID-19 disease also interact with hormonal status. Particularly, lower serum testosterone levels are associated with older age, higher BMI and lower cholesterol levels. Several studies have shown that viral and bacterial infections cause decreased cholesterol levels (56) and that the alterations in lipid levels correlate with the severity of the underlying infection (57, 58). Since cholesterol is the precursor of testosterone, its deficiency also causes testosterone deficiency (59). Actually, a highly significant positive correlation of testosterone with lipoproteins was also found in our cohort. Interestingly, statin therapy, which was frequently encountered in our patients, was found to cause lower total testosterone levels in men with diabetes mellitus (60). However, testosterone levels did not significantly differ between men with or without lipid lowering therapy and also the predictive value of testosterone was independent of concomitant use of lipid lowering drugs.

Testosterone levels are typically decreased in older men (61) due to several factors, including a decline in ability of Leydig cells to produce adequate testosterone in response to LH stimulation (62), which might also partly contribute to the higher COVID-19 related morbidity and mortality within this vulnerable group. Actually, the prevalence of hypogonadism in geriatric hospitalized men over the age of 65 was found to be 53.3 % (63). This is supported by the finding that higher LH/tT ratio was also related to morbidity and mortality. LH levels were within the normal range suggesting the presence of normogonadotropic hypogonadism (64). The absence of compensatory LH secretion in case of low testosterone levels (65) most probably might be caused by altered GnRH and consecutive LH suppression by cytokines as found in patients with immune activation (66, 67). Actually, men were hospitalized in median one week after symptom onset providing a long period for suppression of gonadotropins. The finding that older men over the age of 60 had again lower tT levels compared to young men with concurrent slightly elevated (but still low) LH levels suggests that in older men also the occurrence of late onset hypogonadism might contribute to this rather low tT levels. This is further supported by the positive correlation of the LH/tT ratio with age. Unfortunately, a differentiation of whether these men had age-related Leydig cell dysfunction before COVID-19 or whether low testosterone levels are primarily due to inflammation-related Leydig cell dysfunction or impaired testicular steroid-biosynthesis, cannot be provided by our results.

Testosterone expression can also be suppressed by cortisol which is produced in the adrenal cortex upon stimulation by the adrenocorticotropic hormone (ACTH) secreted by the pituitary gland upon stress-related corticotropin-releasing hormone (CRH) stimulation (68, 69). Activation of the hypothalamic-pituitary-adrenal axis (HPA) in patients with COVID-19 reflected by elevated total serum cortisol levels was shown to be associated with an increased mortality (70) and might also contribute to low testosterone levels in these patients. However, testosterone was also shown to suppress CRH-stimulated cortisol production in men (71) which is why loss of this suppressive mechanism might promote stress-induced HPA activation. Unfortunately, we do not have detected serum hormonal levels of the hypothalamic-pituitary-adrenal axis. Interestingly, hydroxysteroid dehydrogenases, which catalyse steroid biosynthesis (e.g. DHEA to androstenedione, androstenedione to testosterone), is also suggested to be suppressed by immune activation (40).

On the other hand, obesity, linked to disease severity and outcome in SARS-CoV-2 infected patients, is considered to be stringently linked to testosterone deficiency (72). Testosterone can be aromatized to estradiol by the aromatase which is found in abundance in the visceral fatty tissue but also in male gonads (typically in Leydig cells (73), placenta, brain, muscle, bone and vascular tissues (74), and stimulated by cytokines such as IL-6 (75) or Tumor necrosis factor alpha (TNF-α) (76). This is supported by the highly significant negative correlation of inflammatory markers with the tT/E_2_ ratio. Interestingly, estradiol levels were within the normal range thus representing a disturbed relation of testosterone to estradiol primarily caused by quite low testosterone levels rather than increased aromatase activity. Finally, the median BMI was normal. This would also explain the contrary findings in airway epithelial cells in which estradiol was demonstrated to downregulate ACE2 expression (77). Also, recent study results suggest that estrogen receptor signaling is actually protective in mice infected with SARS-CoV-2 (78) by suppressing ACE2 expression as well as pro-inflammatory pathways (79). Th1 immune activation might have stronger impacts on ACE2 expression than estradiol receptor signaling (80). Moreover, lower levels of cardiovascular- and lung-protective ACE2 might also aggravate existing co-morbidities (81). However, higher estradiol level (but within normal range) as well as a lower tT/E_2_ ratio were associated with mortality and morbidity suggesting that this might primarily reflect inflammation-induced aromatase activity, which only slightly increase estradiol levels in men with distinctive testosterone deficiency.

Finally, low DHEA levels upon hospital admission were (similarly to testosterone) also related to higher morbidity and outcome. In vivo experiments showed that DHEA increases macrophage function and promotes a shift of Th1/Th2 balance toward Th1 immunity (82) thus enhancing immunity against viral infection (83). At the same time, DHEA suppresses the expression of various pro-inflammatory cytokines thus preventing overwhelming immune activation (83), suggesting that its deficiency enables overwhelming inflammation with reduced immunity.

### Limitations

This was a retrospective explorative analysis of continuous COVID-19 patients hospitalized at our department. Additionally, sex hormones were not available of all men initially included in the study, which might be an unmeasured bias although baseline characteristics of all men and only those with available sex hormones were almost the same (**Table 1**). This represents a risk for possible type I and II errors. We further have no information about the hormonal status of the patients before their COVID-19 infection which does not allow any conclusion whether these rather low testosterone levels represent hypogonadal hypogonadism or decreased following SARS-CoV-2 related immune activation.

### Conclusion

Our results indicate that men hospitalized due to COVID-19 present with lower testosterone levels showing advanced immune activation and having high risk for an adverse clinical course and a poor prognosis. The origin of testosterone deficiency in these patients might be primarily caused by altered cholesterol biosynthesis in the case of SARS-CoV-2 infection as well as being due to inflammation-induced gonadotrophin suppression. The impact of enzymatic aromatase activation on hypogonadism pathogenesis might be negligible. The latter is supported by the finding that high estradiol levels (but within the normal range) were associated with more severe SARS-CoV-2 infections. Whether these men already had hypogonadotropic hypogonadism before COVID-19 cannot be clarified by our study. Effects of testosterone supplementation on outcome in men with severe SARS-CoV-2 infections should be evaluated in further studies.

## Data Availability Statement

The raw data supporting the conclusions of this article will be made available by the authors, without undue reservation.

## Ethics Statement

The studies involving human participants were reviewed and approved by ethics committee of the Innsbruck Medical University. The patients/participants provided their written informed consent to participate in this study.

## Author Contributions

Conceptualization and methodology, AG and RB-W. Software and formal analysis, LL. Investigation and data curation, AE, FB, GH, LL, LT and RB-W. Resources, AG, AE, FB, GH, GW, MA and RB-W. Writing – original draft preparation, LL. Writing – review and editing, AG, AE, FB, GH, GW, LT, MA, RB-W, G-MP and SK. Supervision, GW. All authors contributed to the article and approved the submitted version.

## Conflict of Interest

Author GH was employed by company MLL Munich Leukemia Laboratory.

The remaining authors declare that the research was conducted in the absence of any commercial or financial relationships that could be construed as a potential conflict of interest.
